# Cardiac magnetic field map topology quantified by Kullback–Leibler entropy identifies patients with clinically suspected myocarditis

**DOI:** 10.3389/fcvm.2023.1276321

**Published:** 2023-11-08

**Authors:** M. Pille, A. Gapelyuk, K. Berg, S. Bannasch, J. Mockler, L.-S. Park, J.-W. Park, N. Wessel

**Affiliations:** ^1^ Department of Physics, Humboldt-Universität zu Berlin, Berlin, Germany; ^2^ Berlin Institute of Health at Charité, Universitätsmedizin Berlin, Berlin, Germany; ^3^ Biomagnetik Park Holding GmbH, Hamburg, Germany; ^4^ Deutsches Herzzentrum der Charité, Campus Benjamin Franklin, Berlin, Germany; ^5^ Department of Human Medicine, MSB Medical School Berlin GmbH, Berlin, Germany

**Keywords:** cardiac magnetic field, Kullback–Leibler distance, myocarditis, magnetocardiography (MCG), linear discriminant analysis (LDA)

## Abstract

**Background:**

Myocarditis is a condition that can have severe adverse outcomes and lead to sudden cardiac death if remaining undetected. This study tested the capability of cardiac magnetic field mapping to detect patients with clinically suspected myocarditis. This could open up the way for rapid, non-invasive, and cost-effective screening of suspected cases before a gold standard assessment via endomyocardial biopsy.

**Methods:**

Historical cardiac magnetic field maps (*n* = 97) and data from a state-of-the-art magnetocardiography device (*n* = 30) were analyzed using the Kullback–Leibler entropy (KLE) for dimensionality reduction and topological quantification. Linear discriminant analysis was used to discern between patients with ongoing myocarditis and healthy controls.

**Results:**

The STT segment of a magnetocardiogram, i.e., the section between the end of the S wave and the end of the T wave, was best suited to discern both groups. Using a 250-ms excerpt from the onset of the STT segment gave a reliable classification between the myocarditis and control group for both historic data (sensitivity: 0.83, specificity: 0.85, accuracy: 0.84) and recent data (sensitivity: 0.69, specificity: 0.88, accuracy: 0.80) using the KLE to quantify the topology of the cardiac magnetic field map.

**Conclusion:**

The implementation based on KLE can reliably distinguish between clinically suspected myocarditis patients and healthy controls. We implemented an automatized feature selection based on LDA to replace the observer-dependent manual thresholding in previous studies.

## Introduction

Myocarditis manifests in a highly heterogeneous manner, from remaining asymptomatic to arrhythmias and cardiogenic shock, leading to sudden cardiac death. The prevalence of myocarditis is therefore believed to be highly underestimated (1). This issue becomes especially pressing in times of a global viral pandemic, affecting a relevant part of the population. In a recent study, Daniels et al. found a prevalence rate of 2.3% in young college athletes after being diagnosed with COVID-19 (2). Only a fraction (five in 37) of these cases would have been detected through cardiac symptoms alone. Therefore, fast and reliable screening is key to preventing long-term negative health effects, reducing the risk of sudden cardiac death, and giving training or resting recommendations for physically active persons.

The gold standard for the diagnosis of myocarditis is still endomyocardial biopsy (EMB), although the American Heart Association, together with other professional societies, recommends performing less invasive methods prior to biopsy to rule out more common causes of cardiac disease (3). Being a non-invasive technique, electrocardiography (ECG) can assess abnormalities in up to 92.6% of all ECG examinations where EMB confirmed myocarditis (4). It is, therefore, still the go-to diagnostic tool for cardiologists when myocarditis is suspected, with the limitation that abnormalities are neither consistent and unique nor necessarily related to the severity of the underlying pathology (3). Another safe and non-invasive tool for diagnosing the inflammatory, edematous, and necrotic manifestations of acute myocarditis is cardiac magnetic resonance imaging (cMRI). By means of cMRI, late gadolinium enhancement can be detected, which was shown to be the best single predictor for all-cause mortality (4).

In this work, we test whether cardiac magnetic field mapping (CMFM) obtained via magnetocardiography (MCG) can function as an additional appliance in the toolbox for myocarditis diagnosis, offering the ability to quickly screen patients and classify between heart-healthy and myocarditis cases. In preceding studies, the primary focus for the application of CMFM was identifying patients with coronary artery disease; a good overview can be found in (5). There, it is concluded that a Kullback–Leibler entropy-based (KLE) approach to quantify the CMFM is preferable over regional parameters due to the inherent dimensionality reduction and less sensitivity to measurement conditions. Other studies investigated the use of CMFM in patients with hypertrophic cardiomyopathy, concluding that KLE is a suitable tool for screening obstructive and non-obstructive manifestations (6).

In addition, a comparison between historic and recent data obtained with a latest-generation first-order gradiometer was performed. The main difference to older devices is the increased number of sensors, resulting in a multi-channel measuring device. This allows measuring the whole chest area in one run compared to multiple runs on older devices. The main advantage of the multi-probe device is the ability to perform quasi-stationary measurements at once and a significant reduction of screening duration and artifacts through movements or residual effects, which is especially useful for detecting transient effects or stress. The underlying technology of low-temperature superconducting quantum interference devices (SQUIDs) is unchallenged because they are still the standard for measuring small magnetic fields. Although other technologies, like optically pumped magnetometers, are tested for the application of magnetocardiography, they do not yet reach a comparable resolution and sensor density (7). Both devices rely on magnetically shielded rooms to reduce noise levels to measure the magnetic field of the heart, which is typical of an order of 10 pT (8).

## Methods

### Magnetocardiographic measurements

The MCG measurements for the different data sets were realized in two different studies, each using a different system, which we will briefly explain.

### System I

The cardiac magnetic field was recorded over the anterior chest wall using a seven-channel magnetic measurement system (Cryoton Ltd., Moscow) based on an LT-SQUID coupled with a second-order axial gradiometer (pickup coil diameter of 20 mm, baseline of 55 mm). The system could be operated in an unshielded environment, but to improve the signal-to-noise ratio, all our measurements were performed in a magnetically shielded room (VAC Akb3b with a shielding factor better than 10,000 at 10 Hz; typical system performance in this environment was 7 fT in a unit band).

**Figure 1:**
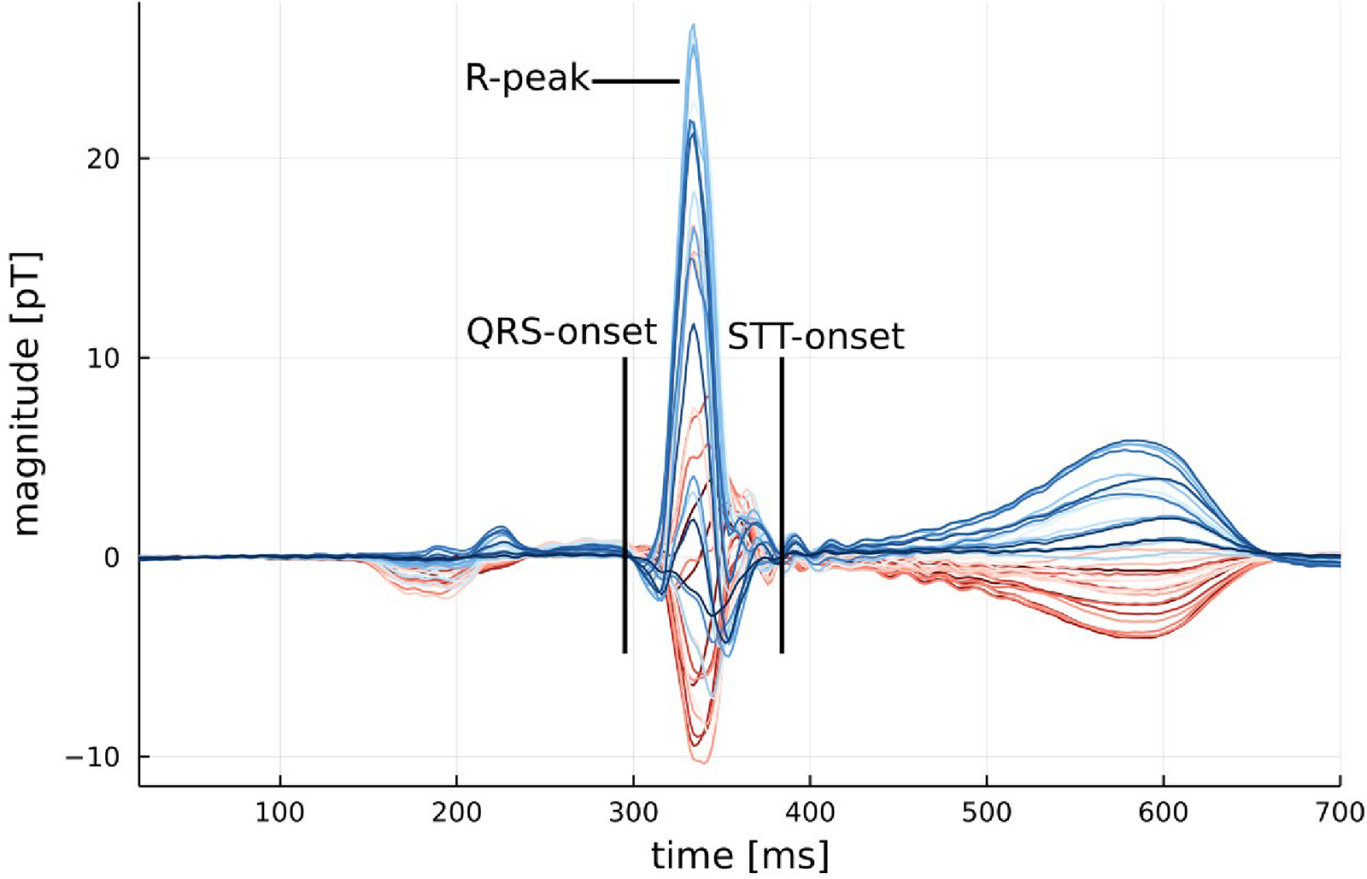
Example of an average MCG signal from a healthy participant with the onset of QRS and STT marked. An example of an average MCG signal for a participant with myocarditis can be found in Figure A2 in the Appendix.

The cardiac magnetic field component perpendicular to the chest wall was measured. CMFM registration was done sequentially at six measurement positions, see Figure 1. ECG lead II was recorded simultaneously for further signal averaging and time alignment. The duration of recording per position was 30 s, with an acquisition rate of 1,000 Hz and a bandwidth of 0.01–130 Hz.

### System II

The system, a CS-Mag II, contains 64 measuring channels evenly distributed inside the cryogenic Dewar with a diameter of 200 mm. Each channel is a first-order axial gradiometer connected to a DC LT-SQUID that measures the *z* component of the cardiomagnetic field perpendicular to the chest. The average noise level of the entire system in a magnetically shielded room is 10 fT in a unit band. The system simultaneously registers ECG in 12 leads. It is also equipped with a non-magnetic bicycle ergometer for stress testing during measurements.

Typical recording time at rest is 2 min with an acquisition rate of 500 Hz. Raw signals were then averaged and baseline-corrected. After that, the signals received from axial gradiometers were recalculated using a proprietary algorithm into another configuration, which is a rectangular grid with signals that would have been received from a system with first-order axial gradiometers.

### Patients

Both studies were approved by the local ethics committee, and informed consent was obtained from each participant. Patients were part of registries for patients with suspected acute coronary syndrome—no patient required mechanical support. Some patients had single premature ventricular or atrial complexes, and none had atrial fibrillation or non-sustained ventricular tachycardia. Magnetocardiographic measurements were performed immediately after admission to the hospital.

### Cohort for system I

Forty patients with acute myocarditis (age 37.3 ± 2.2 years; 28 men, 12 women) were recruited in Franz-Volhard-Hospital (Charite Campus Buch). Patients were admitted to the hospital with typical symptoms: chest pain, dyspnea, fatigue, or palpitations. The diagnosis was based on the following:

1. Laboratory tests with unexplained elevation in troponin and C-reactive protein;
2. Electrocardiographic features of cardiac injury;
3. Wall motion abnormalities in echocardiography;
4. Characteristic cardiac tissue changes in CMRI (e.g., late gadolinium enhancement).

We recruited a group of healthy volunteers from an occupational health center. The 57 healthy volunteers (age 39.6 ± 8.9 years; 35 men and 22 women) had normal findings in echocardiography, bicycle ergometry, ECG, and Holter-ECG. No control participant had a history of cardiac diseases or symptoms.

### Cohort for system II

The second group consisted of 10 consecutive patients (age 50.1 ± 11.9 years, all men, hospital admission December 2011–May 2013) recruited in Asklepios Hospital Hamburg, Germany. All patients came to the cardiology department via the emergency department with chest pain, dyspnea, fatigue, palpitations, or syncope. The patients were part of a registry of patients suspected of acute coronary syndromes. In nine out of 10 patients a coronary artery disease was ruled out via coronary angiography within 24 h. The diagnosis of myocarditis or peri-myocarditis was based on the medical report at the hospital demission, which consisted of the following:

1. Laboratory tests with unexplained elevation in troponin;
2. Echocardiography and cMRI;
3. EMB-guided approach for patients presenting with acute heart failure with shock, high-grade heart block, or symptomatic ventricular tachycardia.

The healthy group of the second cohort consisted of 20 apparently healthy participants (age 53.1 ± 6.9 years, 17 men and 3 women, weight 68.6 ± 10.37 kg, height 173.35 ± 8.7 cm). “Apparently healthy” was defined as participants that fulfilled the following criteria: no clinically manifested disease, no medication, no somatic cardiovascular risk factors, and no acute infection.

### Preprocessing

The main difference between both devices is that the first requires a protocol with six sequential measurements to cover the relevant area, while the second device covers this area in one pass, thus speeding up the examination. The multi-channel sensor array has a concentric arrangement of SQUIDs for the sensor head. Therefore, the geometric distribution of the sensors requires a prespecified adaptation of the lead field matrix to solve the inverse problem.

Data from both devices were transformed into a common format defining a 6 × 6 grid covering an area of about 20 cm × 20 cm at a sampling frequency of 1,000 Hz and a bandwidth of 0.01–130 Hz. Averaging the signal to reduce the signal-to-noise ratio, each magnetic map ***B*** contains 1,000 samples for each of the 36 measurement positions, where each *R* peak is positioned at sample 333. The strength of the magnetic field in each map is then normalized to compensate for a fluctuation in the distance of the measurement device to the heart. The normalization factor (*γ*) is defined as the average field strength during the onset and the end of the QRS complex (*t*_QRS_) as defined in Equation (1):

(1) $$\gamma =\frac{1}{36}\sum_{i=1}^{36}\frac{1}{t_{\mathrm{QRS}}}\sum_{t=\mathrm{QR}S_{\mathrm{onset}}}^{\mathrm{QR}S_{\mathrm{end}}}\left|B_{t}^{i}\right|$$

### Cardiac magnetic field map quantification

The KLE has been proven to reliably quantify differences in the topology of CMFMs. It is a measure of how one distribution differs from another, and in our case, it takes the form of Equation (2), in which *P* denotes the participant's CMFM, *Q* denotes a reference CMFM, which is constructed as the average map of a reference group, and index *i* iterates over all 36 measurement positions:

(2) $$\mathrm{KL}(P,Q)=\sum_{i=1}^{36}P_{i}\cdot \mathrm{Ln}\frac{P_{i}}{Q_{i}}$$

KLE values are calculated for certain segments of interest, which are the 80-ms segment following the onset of QRS and the 250-ms segment from the STT onset, as shown exemplarily in Figure 1. These segments were chosen because they contain the majority of the power in the CMFM and therefore offer the best signal-to-noise ratio. Another argument for selecting these segments can also be made from typical abnormalities found in the ECG associated with myocarditis, a disease that predominantly affects the ventricular myocardium, which are non-specific STT wave changes and PR segment depression (9).

### Statistical analysis

Linear discriminant analysis (LDA) (10) was used to obtain a classification rule for the normalized CMFMs. The robustness of the resulting classification was checked by using a leave-one-out cross-validation (LOOCV), which has been proven to minimize bias and mean-squared error in an LDA (11).

The non-parametric Mann–Whitney *U* test was deployed to assess whether there was a significant difference in the mean heart rate between the two groups (12). This is important as a primary symptom of acute myocarditis is sinus tachycardia, which could have been detected without using an MCG (13).

Preprocessing and statistical analysis were performed using the Julia programming language v1.7.2 (14). The code used for this paper is available on GitHub: https://github.com/AG-CVP/KLE_Analysis.

## Results

### Differences in heart rate and segment length

**Figure 2:**
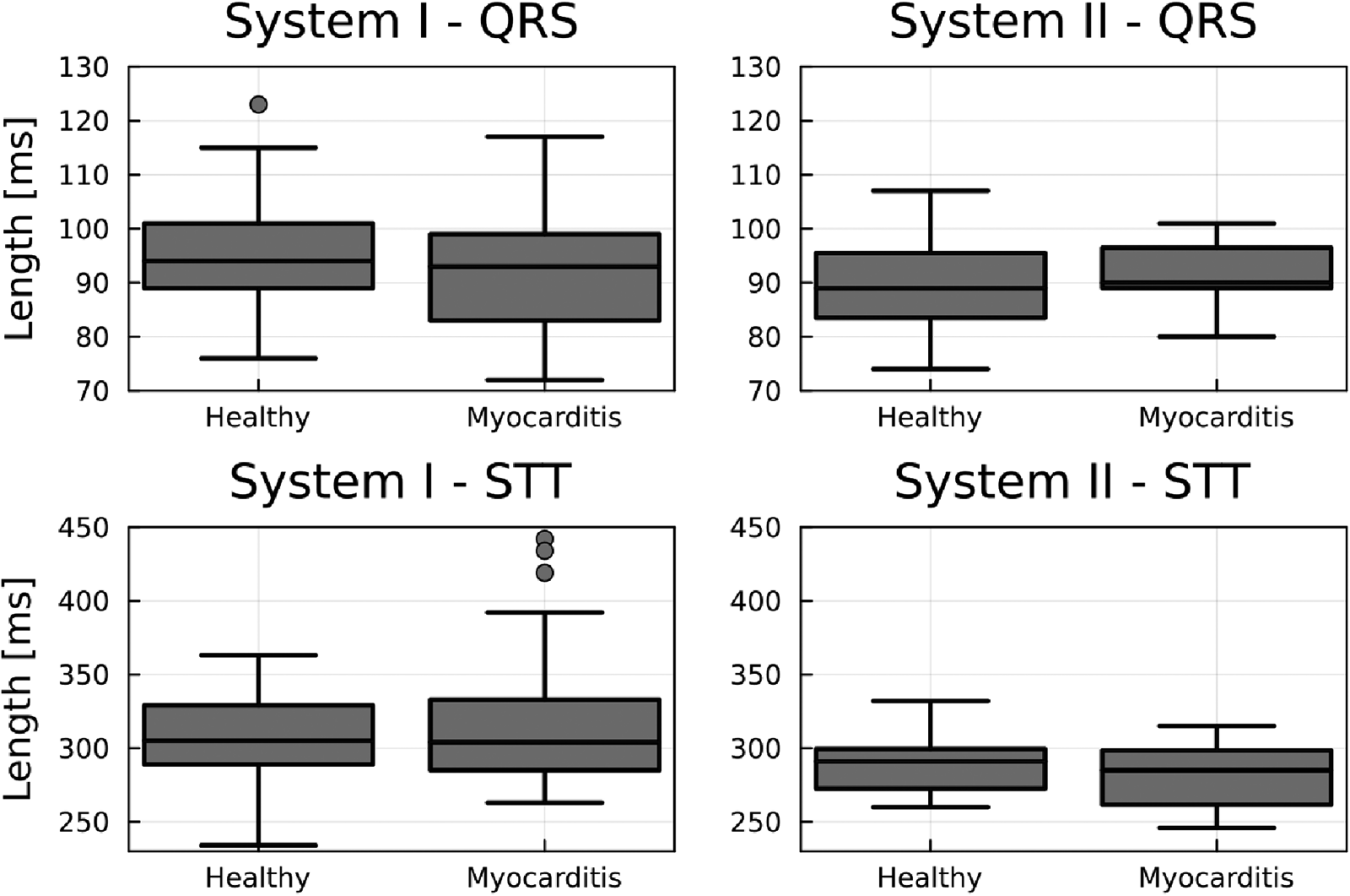
Boxplots comparing the length of the QRS and STT between groups for each device. No significant between-group difference exists. However, there is a relevant intra-group spread and differences between systems for the STT segment.

No significant difference in mean heart rate at rest between the healthy and myocarditis groups was found (*p* > 0.37). Similarly, no significant difference between groups in the length for different segments was found, as displayed in Figure 2, which is an important precondition to excluding the introduction of systematic errors. What can be seen is that the range of segment lengths inside a group is large compared to the average length. The differences between systems, especially for the STT segment, are also non-neglectable. This could lead to the hypothesis that instead of a change in topology, and therefore a change in the underlying physiological process, KLE only quantifies a difference in the segment length.

To counter this hypothesis, we introduced an additional normalization routine to assess the influence of segment length variation. The normalization is realized by resampling each segment, marked by a beginning and end cursor, to segments with an identical number of samples. For this, interpolation between samples was used. The number of samples was then reduced to minimize the correlation between samples across participants to meet the assumptions of the LDA. For the QRS segment, 20 samples were appropriate, and for the STT segment, 40 samples were chosen. This limited the correlation between samples within a segment to below 0.2 but preserved the information on magnetic field distribution.

### Classification of myocarditis through KLE

**Figure 3:**
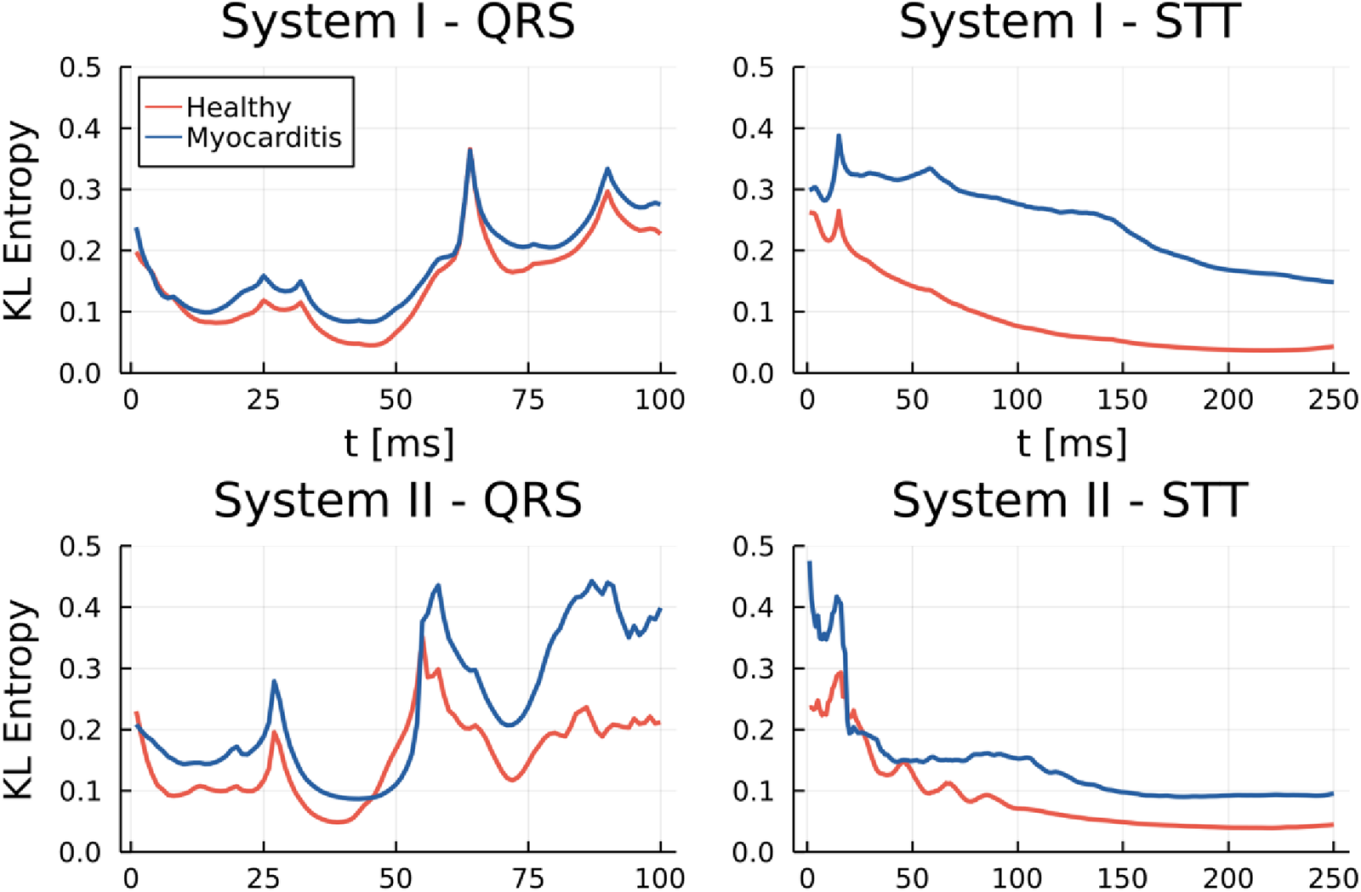
Mean KL entropy for different groups and both devices during QRS and STT.

KLE analysis was performed on the data from both systems I and II for the QRS and STT segments. The reference map was constructed from the healthy group separately for each system. Figure 3 shows the group KL entropy for both segments of interest. A clear difference in the group means is visible for the STT segment, while it is not present in the QRS segment.

Table 1 shows the classification results for segments with a variable length defined by a start cursor. The STT segment results in the best classification for system I, while QRS and STT perform similarly for system II. The joint evaluation of both QRS and STT segments does offer little to no improvement over evaluating the STT segment only.

**Table 1:** LDA classification results by segment and system.

	QRS	STT	QRS + STT
System I	SN: 0.54, SP: 0.73, ACC: 0.68	SN: 0.81, SP: 0.83, ACC: 0.80	SN: 0.81, SP: 0.84, ACC: 0.82
System II	SN: 0.58, SP: 0.80, ACC: 0.71	SN: 0.61, SP: 0.76, ACC: 0.72	SN: 0.69, SP: 0.80, ACC: 0.75

SN, sensitivity; SP, specificity; ACC, accuracy.

An identical analysis performed on the normalized segments, depicted in Table 2, resulted in a comparable sensitivity and specificity for the STT segment and the joined analysis. However, the sensitivity for the QRS segment seems to be improved by the normalization procedure.

**Table 2:** LDA classification results by segment and system for normalized segments.

	QRS—norm	STT—norm	QRS + STT—norm
System I	SN: 0.76, SP: 0.74, ACC: 0.74	SN: 0.81, SP: 0.76, ACC: 0.79	SN: 0.83, SP: 0.85, ACC: 0.84
System II	SN: 0.73, SP: 0.81, ACC: 0.78	SN: 0.75, SP: 0.81, ACC: 0.79	SN: 0.69, SP: 0.88, ACC: 0.80

SN, sensitivity; SP, specificity; ACC, accuracy.

### Healthy participants form a stable cluster

**Figure 4:**
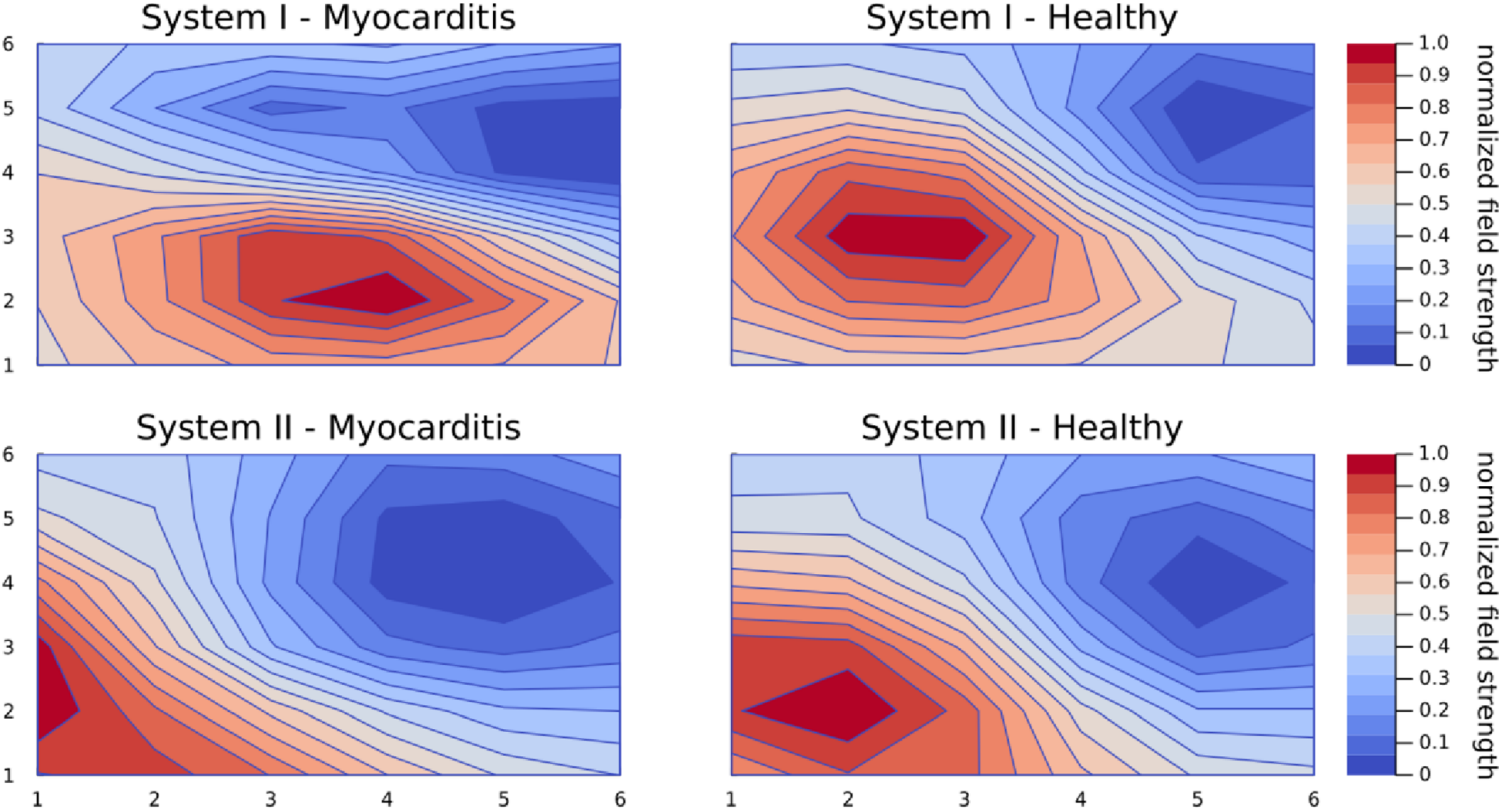
Average magnetic maps for each group and system at the peak of the T wave. This figure exemplifies the phenomenon that maps from healthy controls are very similar even across systems but maps from patients with an underlying pathology are all different in a unique way.

A common observation when comparing CMFMs by means of KLE or just by visual inspection of the averaged MCG is that healthy participants are very similar to each other, but maps with an underlying pathology are different in their unique way. Figure 4 shows the average magnetic maps at the peak of the T wave to illustrate this phenomenon. It can be seen that health maps are similar disregarding the system, while the myocarditis maps are different not only from the healthy maps but also from each other.

**Figure 5:**
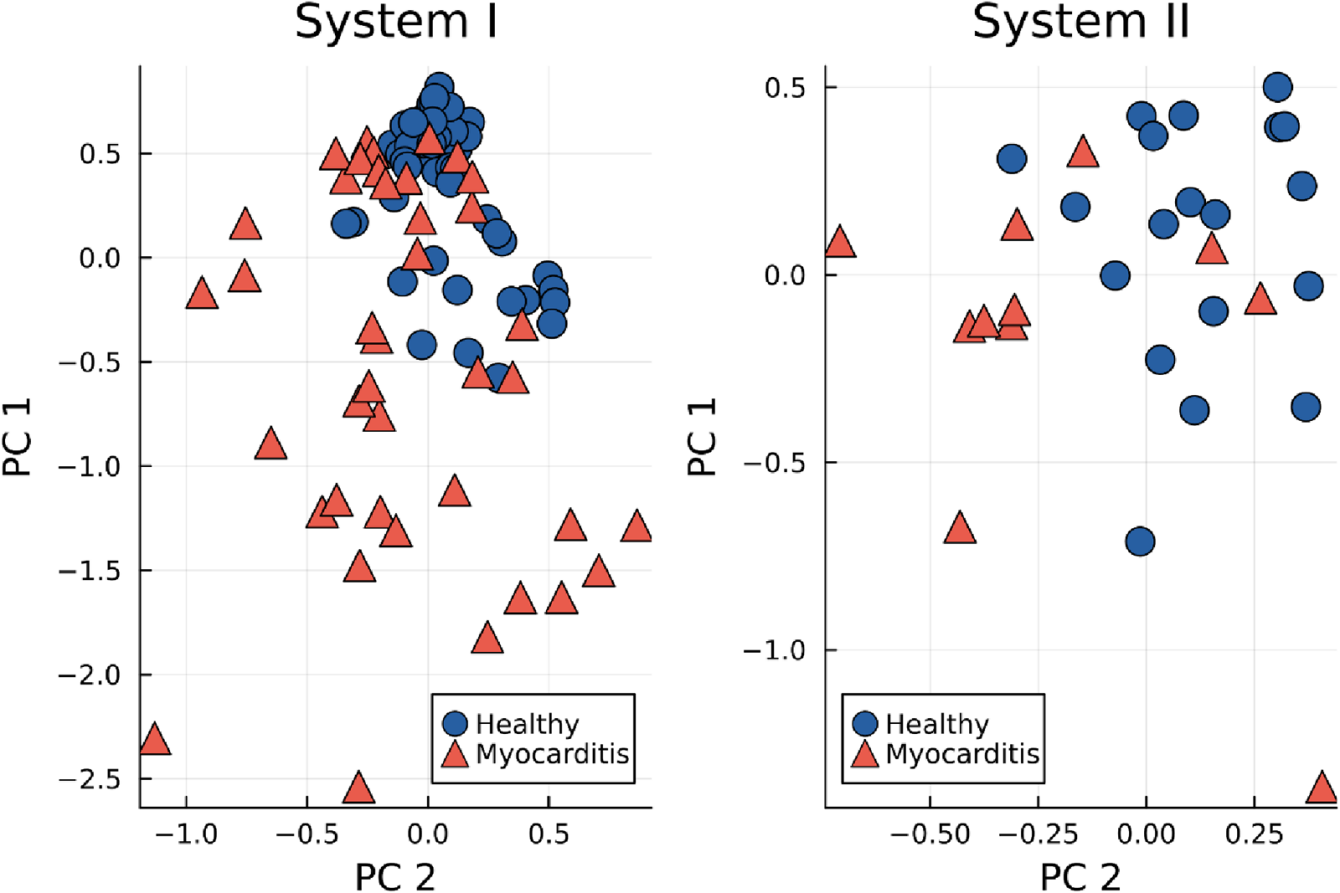
First two principal components (PC) of the KL entropy for the STT segment, grouped by healthy controls (blue dots) and myocarditis patients (red triangles).

For a more systematic look at the clustering of healthy maps, the first two components of the principal component analysis (PCA) for the STT segment are displayed in Figure 5 for both devices. Especially for system I, the healthy group forms a dense cluster, while myocarditis is spread across the first dimensions. For system II, the picture is not as clear, but with the first two dimensions, clusters between both groups become already visible.

**Figure A1:**
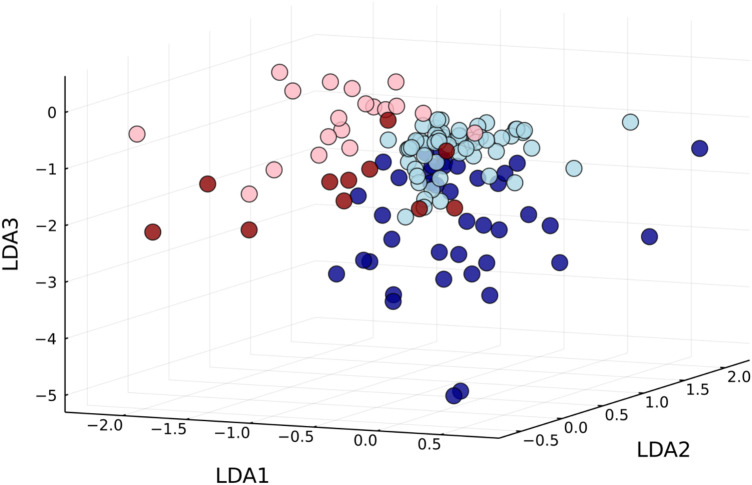
Using a joined reference map, the first three dimensions of the linear discriminant analyses show clusters forming for healthy controls (light) and myocarditis patients (dark), but measurements performed by different systems remain disjoint (system I: blue; system II: red).

If the data from both groups are evaluated with a joined reference map through a single PCA, two dimensions are no longer sufficient to display dense clusters. In three dimensions, clusters start to appear, but healthy clusters from both systems do not completely overlap due to systemic differences (cf. Figure A1 for the first three dimensions resulting from a linear discriminant analysis).

## Discussion

This study investigated the general possibility of magnetocardiography to distinguish between patients with clinically suspected myocarditis and healthy controls based on historical and recent data. The main finding of our analysis was that the CMFM contains reliable information for the classification between myocarditis patients and healthy controls, with accuracies of 0.84 for system I and 0.80 for system II. KLE has proven to be a robust measure for changes in topology even under the presence of variability in segment length (an accuracy of 0.82 for system I and 0.75 for system II), which is in accordance with previous findings.

The retrospective nature of this investigation introduces some limitations, which shall be discussed in a structured form to provide a guideline for future MCG studies.

### System-based arguments

The technology behind MCG measurement is still in the phase of major development. This means that different systems can have fundamental differences in their intrinsic hardware configurations. In this study, one system consists of seven axial gradiometer channels, while the other consists of 64 axial gradiometers, with many more realizations appearing throughout the literature. The same applies to software and clinical routines where no established standard exists. All this is a significant challenge for reproducibility and quality assurance. This problem type arose in several other domains before, and a proven strategy for tackling this is introducing a standard structure for data and meta information. A good example is the brain imaging data structure (BIDS) format used in neuroscience (15) to make sharing data between labs and creating automatic analysis pipelines as smooth as possible. The format was later extended to the specific aspects of magnetoencephalography (16), which could serve as a template for specifying a structure suited for magnetocardiography. We, therefore, express the need for a standardization of MCG data acquisition (17).

A major disadvantage of both systems I and II is the requirement for a shielded room setup to isolate the precise SQUIDS from the environmental noise. Although some procedures are found in the literature that work around using either shielding (18) or SQUIDS (19), all of them require the specified system at hand and are limited to certain regions of the MCG (19). To date, the best overall MCG precision is achieved with shielded SQUID systems, although new systems have been developed and tested in multicenter clinical trials using unshielded magnetocardiography in hospital settings (17).

A further limitation is the individuality of each MCG system since they are created from research facilities or at least built for such. Therefore, it is often hard to compare the hardware settings and internal filters that are used. They can differ in effects like ringing, which is visible in Figure 1, from the start of STT until around 500 ms. Another minor point to discuss is the influence of varying measurement positions between patients, potentially enhanced by unique anatomical features in different patients. The position variation due to placement below the device is usually automatically corrected by centering the r-peak inside the cardiac map.

Although other methods can identify cases of myocarditis, e.g., MRI, they require contrast agents, which can cause allergic reactions and depend on the localization of edema and other signs of myocardial damage, which demand individual interpretation. Furthermore, the MRI causes tissue magnetization due to high magnetic fields, which limits reliable measurement intervals to a minimum break of 2 days. In contrast, recently, Brala et al. (20) presented an MCG-based method only to diagnose myocardial inflammation and monitor treatment response based on a *magnetocardiography vector.* Their method is valuable for an early diagnosis and monitoring response to immunosuppressive therapy in inflammatory cardiomyopathy.

### Methodological arguments

We used the simple approach via KLE together with an LDA, as it is well established in the analysis of CMFMs. However, this approach makes strong assumptions about the data. The first assumption on which the analysis is based is that only a defined physiological process is compared through the comparison between segments of interest. Figure 2 illustrates a significant variability in the duration of these processes. Despite this variability, the classification gave reasonable results for the STT segment (cf. Table 1). A normalization method was introduced to correct for differences in process duration through resampling to examine whether the classification accuracy could be improved, which was the case, especially for the QRS segments (cf. Table 2). Normalization has the advantage that reducing the sampling frequency can reduce the correlation between features to meet the assumptions of the LDA (10). At the same time, hidden potential physiological information gets erased by this procedure. A more exhaustive approach would add the length of each physiological process as a new set of features to the classification, which could be especially important for pathologies with a known influence on the conductivity of the myocardium.

Using our method, myocarditis patients form a stable cluster within the first two principal components (cf. Figure 5). One could argue that these clusters are formed by construction because the KL entropy is calculated against a reference map built from healthy control data. However, on the contrary, when a reference map is created from myocarditis data, neither myocarditis nor healthy clusters appear, and classification results fall close to random choice, indicating that healthy patients are similar, but every myocarditis brings its unique changes. A large database of MCG measurements for a broad range of pathologies could potentially provide more sensitive diagnoses for specific heart diseases through the comparison with different reference maps.

The KL entropy as a statistical distance does not define a metric in the strict mathematical sense, meaning that the distance between distributions A and B does not have to be identical to the distance between B and A. Therefore, absolute KL values are not easily comparable and only valid in a relative context. There exist derived statistical distances that are symmetric and define a metric (21). Future investigations should probably adopt such a methodology for better interpretability. Particularly when dealing with several different systems, a method that allows for comparison of distances is important to accurately assess and correct batch effects.

Our analysis focuses on one procedure: extracting the distance-like measure from KLE after LDA processing of the CMFM for two MCG systems. There are different and broader scopes using machine learning approaches to tackle multiple variables for big data sets at once (18) for cases of ischemic heart disease. This procedure can, in principle, be applied to other pathologies, requires a larger data set, and can potentially discover physiological causes hidden from single-variable analyses and hypotheses. This contradicts the low prevalence of myocarditis and results in a natural limitation.

### Physiological arguments and limitations

The biggest drawback of the comparison between the two system data sets for the cohort for system II was the high variation in possible clinical implications of the included patients. This retrospective analysis was solely based on the diagnosis of myocarditis and peri-myocarditis in medical reports of patients. Data on the LVEF value or echocardiographic wall motion abnormalities were not provided. All patients were selected from the emergency department with symptoms indicating acute coronary syndromes such as chest pain, dyspnea, fatigue, palpitations, syncope, or repolarization abnormalities in ECG. Of 10 patients, nine underwent a coronary angiography within 24 h.

Despite this high intergroup variation between myocarditis patients, the performance of the CMFM evaluation via KLE managed to produce satisfying sensitivity and specificity values for the selected STT segment. Although the QRS segment also showed solid results, especially after normalization, the physiological background of the underlying repolarization still favored the STT segments. The cause of repolarization is ionic currents, which re-establish the action potential necessary for the contraction of the heart. The plateau phase of the action potential is maintained by a delicate balance between incoming Ca^2+^ currents and outgoing K^+^ currents. K^+^ currents dominate, and the membrane potential returns to resting potential. This balance may be disturbed in hypoxia or myocyte damage by myocarditis, leading to heterogeneous repolarization. Usually, high values of troponin are used as a biochemical marker for destroyed myocytes (22).

### Outlook

Our research shows that CMFM can be adapted to indicate patients with clinically suspected myocarditis. The MCG is able to spatially analyze ionic currents of the heart without the need for invasive measures (23). With careful selection of future participants, it may be possible to create a detection of hidden or early stage myocarditis (20). This poses some difficulty for upcoming study designs since a biopsy for healthy participants is out of the question. More analyses are necessary to resolve if a disease-specific separation is possible and can even be expanded to other acute illnesses that affect the heart muscle like long-CoViD. Since some non-treatable heart diseases require well-established early detection, MCG provides a risk-free method for data acquisition.

Technical and computational advances in MCG enable a fast and accessible screening with no negative effects on patients. What is still lacking is broad-scale, long-term evidence for the MCG coupled with insufficient training of clinicians and the unavailability of measurement systems. These challenges could be solved with innovative hardware solutions that improve MCG reliability and offer the possibility to produce reliable and portable instruments tailored to specific clinical applications. However, despite the lack of standardization and the wide variability of MCG instruments, we need to present a prospective validation of our results on a shareable database for large retrospective studies showing the true diagnostic potential of MCG based on statistically robust analyses using all available algorithms for myocarditis detection. These studies would be extremely important for developing novel MCG devices and prospective multicenter clinical trials.

## Figures and Tables

**Figure A2:**
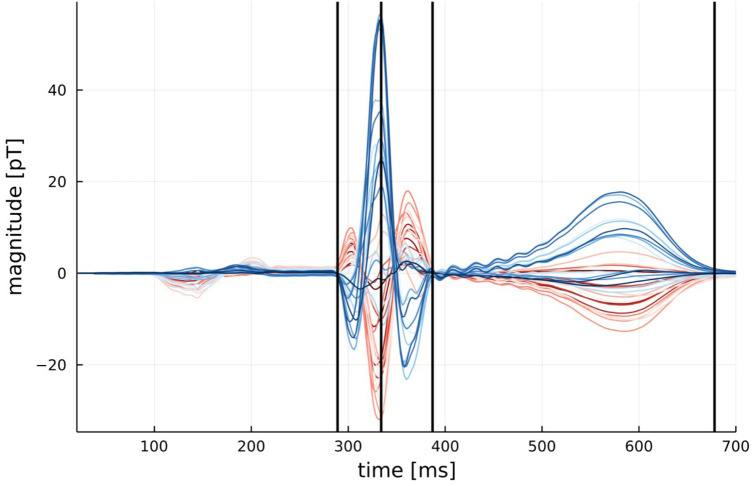
Example of an average MCG signal from a patient with myocarditis. QRS onset, QRS peak, STT begin, and T wave end are marked by black cursers.

## Data Availability

The original contributions presented in the study are included in the article/Supplementary Material, further inquiries can be directed to the corresponding author.
